# Application and development of nanomaterials in the diagnosis and treatment of esophageal cancer

**DOI:** 10.3389/fbioe.2023.1268454

**Published:** 2023-11-03

**Authors:** Qianwen Xiao, Yafei Zhang, Anshun Zhao, Zhikang Duan, Jun Yao

**Affiliations:** Henan Key Laboratory of Cancer Epigenetics, Cancer Institute, The First Affiliated Hospital, College of Clinical Medicine of Henan University of Science and Technology, Luoyang, China

**Keywords:** esophageal cancer, nanoparticles, anti-cancer, diagnosis, treatment

## Abstract

Esophageal cancer is a malignant tumor with a high incidence worldwide. Currently, there are a lack of effective early diagnosis and treatment methods for esophageal cancer. However, delivery systems based on nanoparticles (NPs) have shown ideal efficacy in real-time imaging and chemotherapy, radiotherapy, gene therapy, and phototherapy for tumors, which has led to their recent widespread design as novel treatment strategies. Compared to traditional drugs, nanomedicine has unique advantages, including strong targeting ability, high bioavailability, and minimal side effects. This article provides an overview of the application of NPs in the diagnosis and treatment of esophageal cancer and provides a reference for future research.

## 1 Background

As a common malignant tumor of the digestive system, the global incidence and mortality rates of esophageal cancer rank seventh and sixth among all malignant tumors, respectively. The number of male patients is approximately 2–3 times that of female patients (Sung et al., 2021). Currently, there are two main histological subtypes of esophageal cancer, comprising esophageal squamous cell carcinoma (ESCC) and esophageal adenocarcinoma (EAC), each of which have significant geographical differences and different risk factors. EAC, which is highly prevalent in Western countries, is associated with Barrett’s esophagus, gastroesophageal reflux disease, obesity, and smoking. In contrast, ESCC, which is highly prevalent in China and some other East Asian regions, is associated with smoking, alcohol abuse, and poor dietary habits (Morgan et al., 2022). Early symptoms of esophageal cancer are atypical and can be easily overlooked, leading to the majority of patients being diagnosed at an advanced stage, significantly increasing the difficulty of treatment and the chance of recurrence. Despite advances in tumor treatment research in recent years, as well as the emergence of various new drugs, esophageal cancer lacks the targeted therapy options available for lung cancer, which was higher mutation rates (Melosky et al., 2021). Moreover, unlike renal cancer and malignant melanoma, esophageal cancer does not respond well to immunotherapy (Yoneda et al., 2021). Therefore, chemotherapy remains the cornerstone of treatment for esophageal cancer in clinical practice; however, the accompanying toxic side effects and strong drug resistance should not be underestimated.

The rapid development of nanomedicine undoubtedly brings a ray of hope to this problem. Multiple studies have confirmed that nanoparticles (NPs) show good results in tumor imaging, targeted drug delivery, tumor immunotherapy, and tumor photothermal therapy due to their unique structure and properties. To construct an ideal nanomaterial, it is important to consider not only size, shape, and surface charge, but also hydrophilicity and deformability (Lakshmanan et al., 2021). For example, loading of the polymer material poly lactic-co-glycolic acid (PLGA) with polyethylene glycol (PEG) can improve solubility and enhance cell uptake in the tumor microenvironment, while also reducing immunogenicity and prolonging the circulation time of drugs in the blood, ultimately avoiding the recognition of NPs by the reticuloendothelial system (RES) (Ding and Zhu, 2018). Subsequently, due to the damaged endothelial blood vessels and abnormal lymphatic drainage, more NPs accumulate in tumor tissues, known as the enhanced permeability and retention effect (EPR) (Jhaveri and Torchilin, 2014).

**FIGURE 1:**
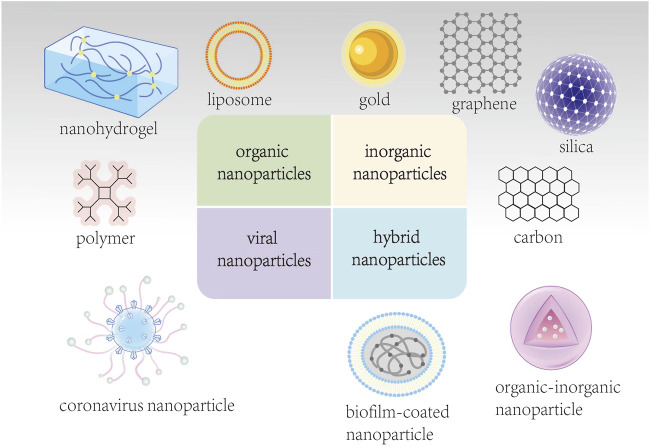
Classification of nanomaterial delivery systems.

Currently, nanomaterial delivery systems used for tumor diagnosis and treatment can be mainly divided into four types (Al-Zoubi and Al-Zoubi, 2022) (Figure 1): 1) organic NPs, such as liposomes, polymers, and nanohydrogels; 2) inorganic NPs, such as gold, carbon, silica, graphene, and other metal NPs; 3) viral NPs (Mellid-Carballal et al., 2023); and 4) hybrid NPs, such as organic-inorganic NPs, and biofilm-coated NPs (Peng and Yao, 2022). This article provides a comprehensive overview of nanomedical research related to esophageal cancer diagnosis and treatment (Table 1), offering different perspectives and a reference basis for future design and exploration of novel nanosystems.

**TABLE 1:** The current application of nanomaterials in esophageal cancer diagnosis and treatment.

	Categories	Examples	Applications	Refs.
Diagnosis	Imaging	SPIO	MRI contrast agents	Motoyama et al. (2012)
		USPIO	MRI contrast agents	Pultrum et al. (2009)
		HB-Au-NPs	Photoacoustic and CT contrast agents	Chen et al. (2021)
		CNFV	CT contrast agents	Gai et al. (2018)
Treatment	Chemotherapy	bMED NPs	Delivery of DOX and β-elemene	Zhan et al. (2020)
		HCS–Dox	Delivery of DOX	Zhang et al. (2017)
		PMPNs	Delivery of DOX and Cur	Gao et al. (2021)
		PEI-Ap-EPI	Delivery of EPI	Wang et al. (2020)
		RGD-fPNPs/EPI	Delivery of EPI and imaging	Fan et al. (2018)
		T7-NP-DC	Delivery of DTX and Cur	Deng et al. (2020)
		DTX-DCMs and BTZ-DCMs	Delivery of DTX and BTZ	Wang et al. (2017)
		FLNP	Delivery of 5-FU and LY	Feng et al. (2018)
	Radiotherapy	GDY-CeO2-miR181a-PEG-iRGD	Radiosensitizer	Zhou et al. (2021)
		iE-PRNPs	Radiosensitizer	Ren et al. (2018)
		USMBs	Radiosensitizer	Shi et al. (2021)
		UiO-66-NH2(Hf)	Radiosensitizer	Zhou et al. (2022)
		^188^Re-liposome	Radiosensitizer	Chang et al. (2015)
	Gene therapy	CEAMB NPs	Delivery of siRNA and DOX	Zhang et al. (2022)
		mEYLNs-Dox/siLPCAT1	Delivery of siRNA and DOX	Jun et al. (2020)
		4WJ-EGFRapt-miR-375-PTX	Delivery of miRNA and PTX	Li et al. (2021)
		miR-203/F-PNDs	Delivery of miRNA and imaging	Deng et al. (2017)
		ZVI@ CMC	DNMTs inhibitor	Hsieh et al. (2022)
		CPNP/shVEGF-yCDglyTK/5-FC	Delivery of suicide gene	Liu et al. (2016)
	phytochemical therapy	GNRs-1/Curc@PMs	Delivery of Cur	Martin et al. (2015)
		rHGFI-Cur	Delivery of Cur	Niu et al. (2020)
		GE11-Ori-Se NPs	Delivery of Oridonin	Pi et al. (2017)
		Ori@GE11-GO	Delivery of Oridonin	Jiang et al. (2018)
	Phototherapy	GCD-Ce6/Pt-EGF	Photosensitizer	Ren et al. (2022)
		CS-GGS	Infrared-induced thermal ablation	Li et al. (2013)
		Fe3O4	Photothermal agents	Chu et al. (2013)
		Cu9S5@MS	Photothermal agents	Wang et al. (2019)
		liposome-BSM	Photothermal agents and SLN mapping	Chu et al. (2016)
		PPy&DOX@TaOx-NIRDye800-PEG	Photothermal agents and triple-modality imaging	Jin et al. (2017)
		PDA NPs	Photothermal agents and Photosensitizer	Zmerli et al. (2021)
		(Au2Se/Au and ZnPc)-loaded BSA nanospheres	Photothermal agents and Photosensitizer	Yu et al. (2013)
		BMIOC	Photothermal agents and Photosensitizer	Liu et al. (2020)
		FA-PPSM	Photothermal agents and Photosensitizer	Chen et al. (2020b)

## 2 Diagnosis

From the histological perspective, the esophagus is a muscular duct that has a certain degree of expansibility and activity. As a result, clinical symptoms of esophageal stenosis or obstruction, such as difficulty in swallowing and pain, only occur when the tumor reaches a relatively advanced local or metastatic stage (Smyth et al., 2017). At this time, endoscopy is undoubtedly the first choice, which not only allows for a biopsy to determine the pathological classification, but also enables the identification of tumor location, lesion length, and surrounding involvement (Schmidlin and Gill, 2021). Imaging examinations, such as computed tomography (CT), magnetic resonance imaging (MRI), positron emission tomography (PET-CT), and single photon emission computed tomography (SPECT), are also used for staging and overall assessment of esophageal cancer. However, these imaging techniques have limitations to varying degrees, including the presence of artifacts, ionizing radiation damage, rapid clearance of contrast agents, and long scanning time (Nagaraju et al., 2021).

To overcome these issues, nanomaterials have been used to develop new methods to optimize tumor imaging (Liu and Grodzinski, 2021). Superparamagnetic iron oxide (SPIO) has been proven to enhance the detection rate of metastatic cervical lymph nodes in patients with esophageal cancer during MRI scans (Motoyama et al., 2012). Furthermore, studies by Pultrum et al. (2009) have shown that ultrasmall superparamagnetic iron oxide (USPIO) combined with MRI can detect most mediastinal and abdominal lymph nodes, providing a significant reference for regional staging and pre-operative assessment. Additionally, a gold nanoprobe labeled with a heterobivalent (HB) peptide ligand, HB-Au-NPs, has demonstrated good results in esophageal cancer imaging, where it can specifically target overexpressed epidermal growth factor receptor (EGFR) and tyrosine kinase receptor 2 (ErbB2) in cancer cells. After injection into xenograft mouse models, a tumor uptake peak can be observed within 8 h, showing strong contrast in photoacoustic and CT imaging, while also exhibiting good stability and biocompatibility (Chen et al., 2021).

Nanomaterials have also received wide attention in the early diagnosis of esophageal cancer. Gai et al. (2018) prepared a novel chitosan-Fe3O4 NP, CNFV, encapsulated with bispecific antibodies against fibroblast growth factor receptor (FGFR) and vascular endothelial growth factor receptor (VEGFR) using a covalent bonding method. The CNFV was used for enhanced CT imaging of patients with suspected esophageal cancer. The results showed that compared to patients who underwent CT imaging alone, CECT-CNFV not only enhanced the resolution of the captured images but also significantly improved the accuracy and sensitivity for diagnosing patients with suspected early stage esophageal cancer. Notably, patients who were diagnosed early with CECT-CNFV had a higher median overall survival and median progression-free survival. As mentioned above, the use of NP-based techniques contributes to the early diagnosis and prognostic evaluation of esophageal cancer, thus guiding personalized treatment.

## 3 Chemotherapy

**FIGURE 2:**
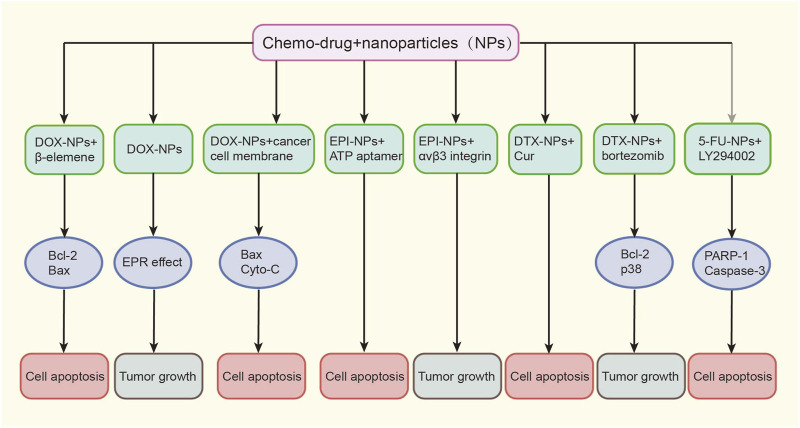
Various nanoformulations of chemo-drugs on EC growth. Doc, docetaxel; EPI, epirubicin; DTX, docetaxel; 5-FU, 5-fluorouracil; EC, esophageal cancer.

Chemotherapy has been used to treat cancer for more than a century. Most chemotherapeutic drugs act on cells during their division phase, inhibiting tumor cell growth by affecting the functions of microtubules, proteins, or DNA synthesis. However, they also exhibit side effects and toxicity to normal human tissues, some of which are even irreversible (Amreddy et al., 2018). Multidrug resistance (MDR) also poses a significant challenge and greatly reduces the effectiveness of chemotherapy (De Jong and Borm, 2008). However, with the help of NPs, chemotherapeutic drugs can achieve targeted delivery (Figure 2), increasing drug bioavailability and improving the therapeutic effects, with an effective cycle time and minimal side effects (Al Bostami et al., 2022).

Doxorubicin (DOX) has been widely used to treat various cancers including esophageal cancer. Liposomal doxorubicin was the first liposome-encapsulated anticancer drug to gain clinical approval (Rivankar, 2014). Research on the combination of DOX and NPs for the treatment of esophageal cancer is ongoing, with the aim to achieve lower drug toxicity and better tolerance. Zhan et al. (2020) used mesoporous silica as a carrier, loaded DOX and β-elemene simultaneously, and absorbed hyaluronic acid on its surface to construct a dual-drug co-delivery nanosystem termed bMED NPs. Through a series of *in vitro* and *in vivo* experiments, bMED NPs have been proven to have not only good antitumor effects, but also accumulate in tumor tissues to achieve drug release and prolonged circulation in the body. Zhang et al. (2017) developed novel hollow carbon spheres (HCSs) for the efficient delivery of DOX. Their results showed that these spheres were easily engulfed by cancer cells and unaffected by environmental pH. Moreover, due to their ability to alter drug distribution and prolong circulation time, they exhibited significant inhibitory effects on esophageal cancer cells. Additionally, a nanocomposite composed of the polymer material PLGA connecting DOX and curcumin has been used for the treatment of drug-resistant esophageal cancer cell lines after being wrapped by TE10 cell membranes. Both *in vitro* cell studies and *in vivo* animal models have demonstrated the strong targeting and anti-tumor activity of these NPs, which also showed good biocompatibility, effectively avoiding the side effects of chemotherapeutic drugs and providing a novel strategy to treat multidrug-resistant esophageal cancer (Gao et al., 2021).

Epirubicin (EPI) is an isomer of DOX, which has received favorable attention from many researchers. In a study conducted by Wang et al. (2020), EPI was first inserted into an ATP adapter (Ap) to form a double-stranded DNA, before being compressed using polyethyleneimine (PEI) to successfully construct the PEI-Ap-EPI nanosystem. After being ingested by esophageal cancer cells, PEI-Ap-EPI was shown to open and release EPI in an ATP-enriched environment within the cells, significantly improving the inhibition efficiency against cancer cells. Fan et al. (2018) also proposed and synthesized fluorescent peptide-assembled NPs (f-PNPs) for the delivery of EPI and imaging of tumor tissues. To achieve tumor targeting, f-PNPs were first conjugated with the RGD peptide to selectively target esophageal cancer cells through the αvβ3 integrin, before embedding the NPs with EPI to obtain RGD-fPNPs/EPI. This system not only demonstrated the enhanced EPR effect of RGD-fPNPs/EPI at the cellular level, resulting in greater EPI accumulation in tumor tissue, but also proved the targeted delivery and tracking of EPI *in vivo* using a xenograft mouse model, achieving drug delivery to the tumor site and facilitating monitoring of the treatment response with the help of NIR fluorescence.

As a kind of natural cytotoxic drug, the taxane family changes the carcinogenic process of multiple cells, including mitosis, angiogenesis, inflammatory reaction, and cell apoptosis (Mosca et al., 2021), and its second-generation product docetaxel (DTX) is a frontline drug for treating esophageal cancer. To further improve the efficacy of DTX, Deng et al. (2020) loaded DTX and Cur together onto NPs to form T7-NP-DC. T7 functioned to enhance the targeted distribution of the nanomedicine to tumor tissues and significantly increased the release of DTX and Cur through the pH response, effectively exerting a safe and efficient anti-tumor effect. Additionally, Wang et al. (2017) prepared nanoscale formulations of DTX and bortezomib (BTZ) using a disulfide-bond-crosslinked micelle (DCM) platform (DTX-DCMs and BTZ-DCMs), further revealing that NPs loaded with chemotherapeutic drugs not only have the advantages of a small diameter and high loading rate, but can also be effectively internalized by esophageal cancer cells to induce apoptosis in a dose-dependent manner.

The 5-fluorouracil (5-FU) is another commonly used chemotherapy drug for treating esophageal cancer. The 5-FU works by inhibiting the synthesis of thymidylate synthase, thus blocking DNA synthesis and ultimately affecting tumor growth and proliferation. However, toxicity and MDR remain major factors limiting its effectiveness (Longley et al., 2003). Previous studies have reported that autophagy inhibitors can overcome the MDR of cancer cells (Kumar et al., 2012). Therefore, Feng et al. (2018) designed a PEG nano-liposome loaded with both 5-FU and the autophagy inhibitor LY294002 (LY) for targeted therapy of esophageal cancer. The results confirmed that this unique drug delivery system achieved controlled release of both components, with a relatively faster release rate of LY compared to 5-FU. Moreover, when autophagy was inhibited, the cancer cells showed significantly increased sensitivity to 5-FU, leading to higher levels of apoptosis.

## 4 Radiotherapy

Radiation therapy is a commonly used method for treating esophageal cancer. Concurrent chemoradiotherapy (CCRT) is of vital importance for patients with inoperable or pre-operative esophageal cancer, while radiation therapy alone provides a good choice for patients who cannot tolerate or have shown a limited response to chemotherapy (Li et al., 2016). However, due to the presence of intrinsically and radiation-induced resistant tumor cells, radiation therapy is not completely effective. Radiation resistance is mainly caused by hypoxia, DNA damage repair, cell cycle arrest, related gene alterations, and tumor stem cells, and in certain cases, it can even promote tumor invasion and metastasis (Chen et al., 2020a). Therefore, it is crucial to research and develop safe and effective radiosensitizers.

The level of miR181a in the serum of patients with esophageal cancer can be used to predict the sensitivity of locally advanced ESCC to radiotherapy and has a radiosensitizing effect (Xiang et al., 2014). Zhou et al. (2021) designed a multifunctional nanocomposite material to effectively deliver miR181a to tumor tissue. They first anchored CeOs NPs to two-dimensional graphdiyne (GDY) with sp2-and sp-hybridized carbon atoms to enhance its catalase (CAT) activity, and then connected miR181a to it. Finally, they encapsulated the above composite material with PEG-iRGD to improve its dispersibility and stability. When combined with radiotherapy, GDY-CeO_2_-miR181a-PEG-iRGD showed significant radiosensitizing effects in both cell-derived xenograft and PDX (patient-derived tumor xenograft) models of ESCC. Moreover, Ren et al. (2018) took advantage of the common high expression of EGFR in esophageal cancer and coupled the recombinant protein anti-EGFR-iRGD to the surface of PTX wrapped in red blood cell membranes to form a new nanodrug termed iEPRNPs. *In vitro* experiments showed that compared to free PTX, iE-PRNPs had significantly enhanced radiosensitizing effects in EGFR-overexpressing esophageal cancer cells and exhibited good targeting, high penetration, and ideal sustained release characteristics.

Microbubbles (MBs) are materials composed of gas as a core (usually perfluorocarbon) and lipids, polymers, or proteins as a shell membrane, which can be used as a contrast agent for ultrasound imaging after intravenous injection (Czarnota et al., 2012). Ultrasound-stimulated microbubbles (USMBs) have been proven to induce endothelial cell apoptosis to achieve anti-angiogenesis effects. USMBs have also shown synergistic effects with radiotherapy for treating various malignant tumors, such as colon cancer, breast cancer, and prostate cancer (Huang et al., 2013; Al-Mahrouki et al., 2014; Lai et al., 2016). Similarly, the enhanced effect of radiotherapy mediated by USMBs has been observed in esophageal cancer, which was accompanied by the inhibition of proliferation, migration, and invasion ability of esophageal cancer cells, as well as the promotion of their apoptosis (Shi et al., 2021).

Metal nanomaterials also exhibit good radio enhancement effects. Zhou et al. (2022) synthesized UiO-66-NH2 (Hf) with a diameter < 100 nm based on metal-organic frameworks (MOFs) under atmospheric pressure conditions, which maintain stability in physiological environments. *In vivo* and *in vitro* experiments demonstrated that UiO-66-NH2 (Hf) enhanced X-ray absorption leading to DNA breakage in cancer cells, increased generation of reactive oxygen species (ROS), and ultimately induced apoptosis. Furthermore, Chang et al. (2015) used rhenium-188 (188Re) liposomes in combination with radiotherapy for treating esophageal cancer. The results showed that the inhibition rate of tumor growth achieved by this combination therapy reached 53%, which was much higher than that achieved with individual treatments, without increasing biotoxicity and side effects.

## 5 Gene therapy

The siRNA is a type of double-stranded RNA molecule consisting of 20–23 nucleotides (Alshaer et al., 2021). In 1998, by conducting an antisense RNA inhibition experiment in the nematode *Caenorhabditis elegans*, Fire et al. (1998) discovered that double-stranded RNA was 10 times more efficient at inducing gene silencing compared to single-stranded or antisense RNA. The siRNA was speculated to play a central role in RNA silencing in a phenomenon termed RNA interference (RNAi). As a result of this discovery, Andrew Z. Fire and Craig C. Mello were awarded the Nobel Prize in Physiology or Medicine in 2006 (Dong et al., 2019). Since then, siRNA-based therapeutic strategies have been widely used in the research of various diseases, including cancer, viral infections, and genetic inherited diseases. However, the application of siRNA *in vivo* poses challenges due to its instability, off-target effects, and susceptibility to degradation by nucleases (Nikam and Gore, 2018). The emergence of nanodelivery systems undoubtedly provides new solutions to this problem. Zhang et al. (2022) designed a multifunctional carboxymethyl chitosan-based NP (CEAMB NPs) capable of simultaneously delivering DOX and siRNAs targeting major vault protein (MVP) and B-cell lymphoma-2 (BCL2) to explore its anti-tumor effect in esophageal cancer. The results showed that CEAMB NPs not only effectively targeted the tumor site and suppressed the expression of target genes but also greatly enhanced the ability of DOX to induce apoptosis in tumor cells, significantly improving the therapeutic effect. Moreover, Jun et al. (2020) developed mEYLNs-Dox/siLPCAT1, a liposome nanocarrier with a layer of white blood cell membrane wrapped on its surface carrying both DOX and siRNA targeting the lipid synthesis metabolic gene LPCAT1. Studies have shown that this combination of chemotherapy and gene therapy exhibited a strong synergistic effect, achieving a therapeutic effect greater than the sum of individual treatments, while the encapsulation of the white blood cell membrane significantly prolonged the circulation time of the drugs, making it an ideal medium for targeted delivery.

**FIGURE 3:**
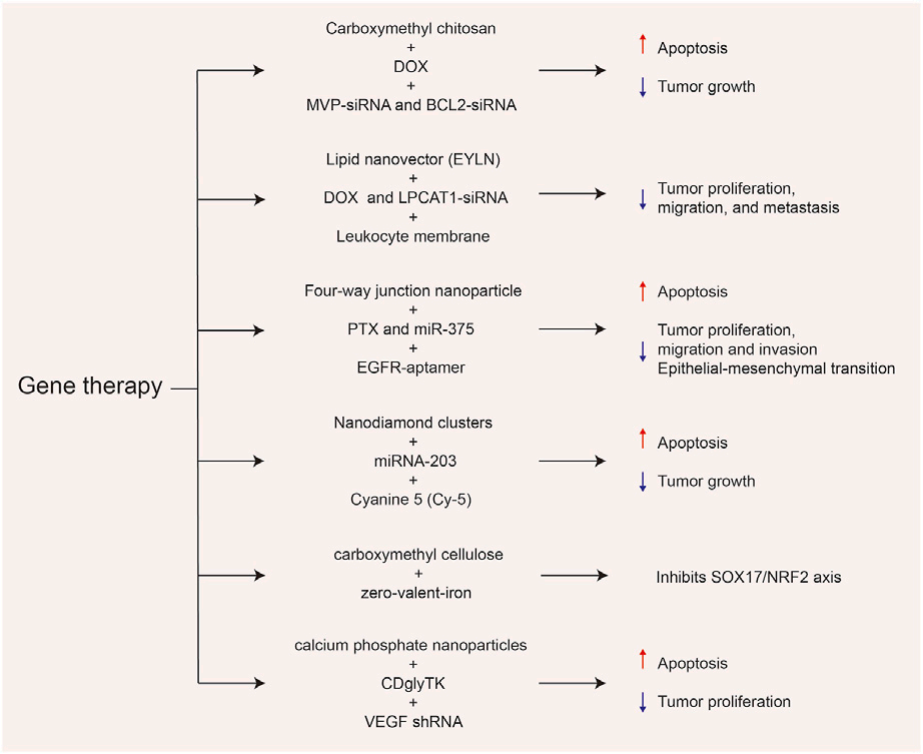
Various gene-related nanosystems and their therapeutic role in esophageal cancer (EC). DOX, doxorubicin; MVP, major vault protein; BCL2, B-cell lymphoma-2; PTX paclitaxel; NRF2, Nuclear factor-erythroid 2-related factor 2; VEGF, vascular endothelial growth factor.

As a type of single-stranded non-coding RNA, microRNAs (miRNAs) play a key role at the post-transcriptional level, participating in the regulation of a series of biological activities *in vivo*, including cell growth, tissue differentiation, and angiogenesis (Diener et al., 2022). The miRNAs are also abnormally expressed in various cancers and regulate the growth, invasion, and metastasis of tumors through functions similar to oncogenes or tumor suppressor genes (Ganju et al., 2017). Therefore, miRNAs have become important therapeutic targets for various cancers. The miR-375 is a tumor suppressor factor that is lowly expressed in ESCC tissues. Increased levels of miR-375 induce the expression of Bax, Caspase-3, and E-cadherin, thereby limiting the occurrence and development of esophageal cancer by promoting apoptosis and inhibiting epithelial-mesenchymal transition (EMT). To facilitate targeted delivery of miR-375, Li et al. (2021) designed a novel four-way junction RNA nanocarrier and loaded it with paclitaxel (PTX) and EGFR-specific aptamer (EGFRapt) to form a 4WJ-EGFRapt-miR-375-PTX nanosystem. Under the modification of EGFRapt, esophageal cancer cells showed significantly enhanced endocytosis of the nanodrug, allowing selective accumulation of miR-375 and PTX at the tumor site, demonstrating stronger therapeutic effects and lower systemic toxicity (Figure 3). Furthermore, Deng et al. (2017) used a simple two-step assembly method to connect the tumor suppressor miRNA-203 and near-infrared (NIR) fluorescent imaging agent cyanine-5 (Cy-5) on the basis of nanodiamond clusters (NDs). They further confirmed that this nanosystem (miR-203/F-PNDs) not only significantly inhibited the proliferation of Ec-109 cells *in vitro* but also achieved precise imaging of the tumor site after intravenous injection for 24 h.

Nuclear factor-erythroid 2-related factor 2 (NRF2), derived from the redox system, is the main regulatory factor in the cellular antioxidant response, with increasing evidence suggesting that NRF2 plays a role in the progression, metastasis, and therapeutic resistance of various cancers (Rojo de la Vega et al., 2018). Hsieh et al. (2022) found that in ESCC, NRF2 was negatively correlated with the expression of a tumor suppressor factor, SOX17, which is lowly expressed due to promoter hypermethylation. They also proved that SOX17 acted as an upstream inhibitor of NRF2 and that its downregulation was associated with a poor response to CCRT and low survival rate. Based on this, they designed carboxymethyl cellulose-coated zero-valent-iron (ZVI@CMC) NPs to inhibit the activity of DNA methyltransferases (DNMTs), thereby restoring the expression of SOX17 and overcoming the tumor resistance caused by NRF2 upregulation. Coincidentally, in order to treat carboplatin-resistant ovarian cancer, researchers have also designed a hyaluronic acid (HA)-decorated metal-organic framework (MOF) to specifically deliver GSK-J, the JMJD3 demethylase inhibitor. The negatively charged HA and positively charged MOF can form a stable nano-shell through strong surface affinity. It has been verified that the formed nanosystem HA@MOF@GSK-J1 can target multiple receptors, such as CD44 and HER2, to treat carboplatin-resistant ovarian cancer cells (Yang et al., 2022).

Suicide gene therapy is a novel approach to treating malignant tumors, in which genes from bacteria or viruses are introduced into target cells, and the enzymes they express are used to activate drug precursors or encode toxic substances for direct gene therapy. This approach induces tumor cell suicide without affecting normal cells (Navarro et al., 2016). Currently, cytosine deaminase (CD) and herpes simplex virus thymidine kinase (HSV-TK) are the two most extensively studied suicide genes (Uckert et al., 1998). Previous studies have shown that the fusion gene (CDglyTK) of yeast cytosine deaminase (yCD) and thymidine kinase (TK) was more effective than a single suicide gene. Therefore, Liu et al. (2016) constructed calcium phosphate NPs (CPNP) to deliver CDglyTK and shRNA against vascular endothelial growth factor (VEGF). CPNP efficiently delivered CDglyTK into EC9706 cells, converting 5-fluorocytosine (5-FC) to the cytotoxic drug 5-fluorouracil (5-FU) locally, leading to death of surrounding tumor cells. Meanwhile, VEGF-targeted shRNA further enhanced the antitumor effect, demonstrating a more effective anti-esophageal cancer effect both *in vitro* and *in vivo*.

## 6 Phytochemical therapy

In recent years, researchers have gradually discovered that certain natural chemical substances found in plants have anticancer activities, which can affect the biological activities of tumor cells through targeting signaling pathways, promoting apoptosis, blocking cell cycle progression, and regulating antioxidant reactions (Jang and Lee, 2023). However, their clinical applications are limited due to issues such as low solubility, poor permeability, high hepatotoxicity, and unsuitable pharmacokinetic parameters. To address these problems, NPs have been used to deliver plant chemical substances to specific tumor cells or tissues, while also improving their water solubility and bioavailability (Rizwanullah et al., 2018).

Cur is a yellow pigment extracted from the rhizomes of the ginger plant, a member of the ginger family. As a lipophilic polyphenol, Cur exhibits anti-inflammatory, anti-cancer, antiviral, and antioxidant properties (Kotha and Luthria, 2019). Martin et al. (2015) used polylactic-co-glycolic-co-polyethylene glycol acid (PLGA-b-PEG-COOH) to simultaneously load Cur and lipophilic gold nanorods (GNRs), forming a new nanosystem termed GNRs-1/Curc@PMs, and verified its anti-proliferative effect in esophageal adenocarcinoma OE-19 cells. Additionally, to overcome the poor water solubility of Cur, Niu et al. (2020) prepared Cur NPs based on class I hydrophobin recombinant HGFI (rHGFI) using a freeze-drying method. The results showed that with the help of rHGFI, the solubility and stability of Cur significantly improved, and it exhibited stronger anti-esophageal cancer effects than the free Cur.

Oridonin is another well-known phytochemical agent with potential anti-cancer activity. An increasing number of studies have demonstrated that oridonin can play a role for treating various malignant tumors, including lung cancer, cervical cancer, breast cancer, gastric cancer, and colorectal cancer (Liu X. et al., 2021). Pi et al. (2017) synthesized selenium element-based NPs conjugated with the EGFR-binding peptide GE11 to encapsulate and deliver oridonin to treat esophageal cancer. GE11-Ori-Se NPs taken-up by KYSE-150 cells (an EGFR-overexpressing esophageal cancer cell line) were found to not only accumulate in lysosomes and release oridonin, but also induce cancer cell apoptosis through inducing ROS production, activating the mitochondrial-dependent pathway, and inhibiting the EGFR-mediated PI3K/AKT and Ras/Raf/MEK/ERK pathways. Similar results were observed in the study by Jiang et al. (2018), where they functionalized graphene oxides (GO) with the GE11 peptide to create a novel nanomedicine (Ori@GE11-GO) for targeted delivery of oridonin. This modification of the GE11 peptide allowed oridonin to achieve specific recognition of EGFR-overexpressing esophageal cancer cells and improve anticancer efficiency through pathways such as cell cycle blockade, disruption of mitochondrial membrane potential, and activation of apoptosis signaling pathways.

## 7 Phototherapy

**FIGURE 4:**
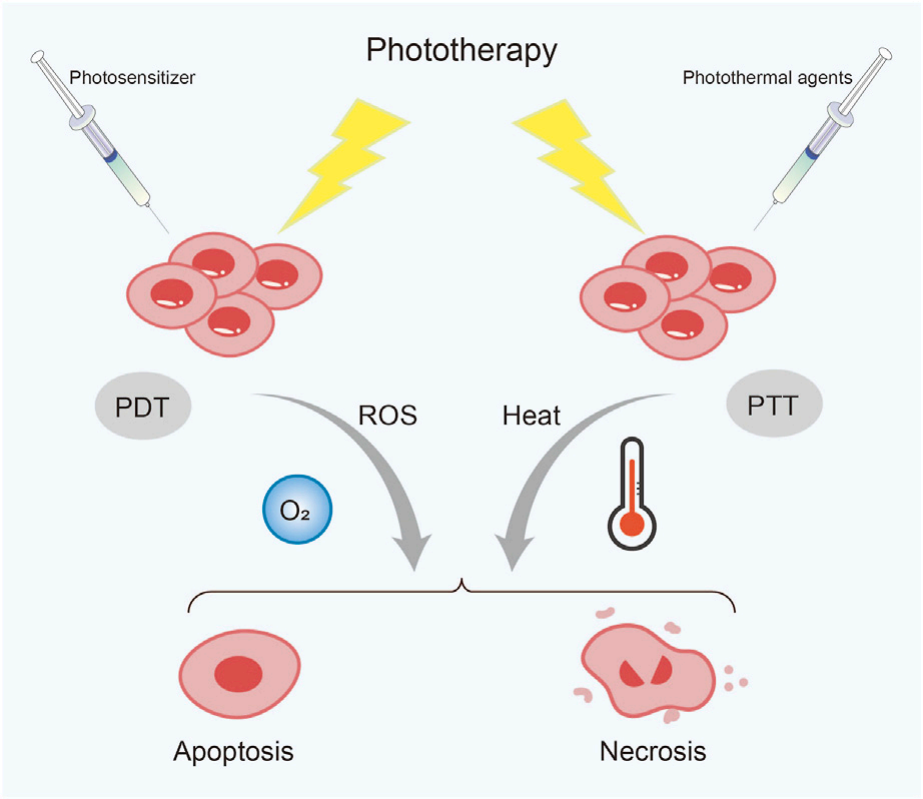
Classification of phototherapy. PDT, photodynamic therapy; PTT, photothermal therapy; ROS, reactive oxygen species.

Recently, phototherapy has attracted much attention from researchers due to its minimally invasive and repeatable advantages, which have led to its gradual use for treating various malignant tumors. Phototherapy includes photodynamic therapy (PDT) and photothermal therapy (PTT) (Li et al., 2020) (Figure 4). PDT mainly relies on the cell death and apoptosis induced by ROS produced by photosensitizers under laser irradiation. The combination of photosensitizers and nanomaterials can improve the efficiency of PDT and reduce toxic side effects (Kwiatkowski et al., 2018). Importantly, this therapy can also help drugs effectively cross the blood-brain barrier (BBB) sand has broad prospects in the treatment of some brain tumors. Mo and his team designed both nanosystems to help the drug better target glioblastoma. One is a combination of yolk-shell nanoparticles, cyclic arginine-glycine-aspartate, and hydroxychloroquine, encapsulated by cancer cell membranes (Mo et al., 2022). The other is exosome-modified combination of zinc sulfide (ZnS), iRGD peptide and hydroxychloroquine (Liu et al., 2023). Under laser irradiation, both types of NPs demonstrate excellent ability to target glioblastoma cells. Hydroxychloroquine effectively inhibits autophagy, significantly improving the therapeutic effect and reducing systemic toxicity. Similarly, PDT also exhibits promising therapeutic effects in esophageal cancer. Ren et al. (2022) used green fluorescence carbon dots (GCDs), which have excellent optical properties as drug carriers, and simultaneously loaded the photosensitizer Ce6, chemotherapeutic drug cisplatin, and targeting ligand EGF to prepare GCD-Ce6/Pt-EGF nanocomposites. Through *in vitro* and *in vivo* experiments, these NPs were found to actively target EGF, avoiding non-specific uptake by normal tissues, increasing the content of Ce6 and cisplatin at the tumor site. Moreover, under 660 nm laser irradiation, these NPs exhibited stronger induction of apoptosis and a stable treatment effect.

PTT is a treatment method that uses the photothermal effect of photothermal agents (PTAs) to convert an external light source, usually near-infrared light (NIR), into heat energy, causing local high temperatures that kill tumor cells (Zhao et al., 2021). Therefore, the selection of PTAs with high photothermal conversion efficiency and good biocompatibility are prerequisites for PTT. Various types of nanomaterial-based PTAs have shown great potential and prospects in tumor PTT, currently mainly classified into five categories: noble metal materials, carbon-based materials, transition metal-based materials, organic small molecule materials, and semiconductor polymer materials (Chen et al., 2022).

Gold nanomaterials do not have inherent therapeutic effects, but their surface plasmon resonance (SPR) characteristics allow them to convert the oscillation of electrons on the particle surface into heat under NIR light irradiation, thereby triggering photothermal effects (Liu XY. et al., 2021). Li et al. (2013) used chitosan coated gold/gold sulfide (CS-GGS) NPs to treat EAC through endoscopic NIR irradiation. The results showed that this treatment method could selectively target cancer cells to achieve therapeutic effects without harming normal esophageal tissue. Chu et al. (2013) also demonstrated that Fe_3_O_4_ NPs significantly inhibited the viability and tumor growth of esophageal cancer cells both *in vitro* and *in vivo* after 808 nm NIR irradiation. Similarly, Wang et al. (2019) coated Cu_9_S_5_ NPs with silica to form a Cu_9_S_5_@MS core-shell nanostructure and investigated its anti-cancer activity on esophageal cancer EC109 and TE8 cell lines. The results showed that Cu_9_S_5_@MS could inhibit the growth of esophageal cancer cells by promoting apoptosis after 808 nm NIR irradiation and exhibited good affinity and biocompatibility. Some natural substances have also shown good photothermal conversion performance in photothermal therapy for esophageal cancer. Indeed, Chu et al. (2016) reported that natural black sesame melanin (BSM) extracted from black sesame could be assembled into 20–200 nm flaky NPs in aqueous solution, while a liposome layer was encapsulated on the surface of BSM to improve its water solubility. In a mouse model with Eca-109 cell-induced esophageal cancer, liposome-BSM not only absorbed NIR light and rapidly converted it into heat energy to achieve therapeutic effects on esophageal cancer, but also accurately located the sentinel lymph node (SLN), providing a reference for the surgical treatment of esophageal cancer.

Despite these successes, single PTT therapy still has limitations. To further enhance the treatment effect, many researchers are attempting to co-load chemotherapeutic drugs and PTAs onto NPs to achieve synergistic therapy of chemotherapy and PTT. For example, Jin et al. (2017) simultaneously encapsulated DOX and polypyrrole (PPy) in the core of hollow tantalum oxide (TaOx) NPs, before coupling NIR fluorescent dye (NIRDye800) onto their shells to form the PPy&DOX@TaOx-NIRDye800-PEG nanosystem. As tantalum has a similar X-ray attenuation coefficient to gold and higher than iodine, it can provide strong contrast in CT imaging, facilitating initial tumor localization. When treating KYSE30 tumor-bearing mice, the NPs significantly increased the temperature of the tumor site after laser irradiation, achieving a tumor growth inhibition rate of up to 100%, with no cases of recurrence. They also demonstrated good photoacoustic and fluorescence imaging capabilities, which assisted with real-time monitoring of tumor location and anatomy. The combination of these three imaging modalities provides new directions for early diagnosis and precise treatment of tumors.

In many PTT-based combination therapies, the combination of PDT and PTT also exhibits a synergistic effect of “1 + 1 > 2”. Zmerli et al. (2021) took advantage of the good photothermal conversion performance of polydopamine (PDA) to conjugate photosensitizers and synthesize polyethylene glycolated PDA NPs. By combining two phototherapy modalities under different wavelengths, a synergistic phototoxic effect was achieved, which significantly inhibited the growth of esophageal cancer KYSE-30 and HET-1A cells. Additionally, Yu et al. (2013) prepared a nanocomposite of thermosensitizer gold selenide (Au_2_Se/Au) and photosensitizer zinc phthalocyanine (ZnPc), which was irradiated with a 655-nm laser to generate ROS and thermotherapy effects on esophageal cancer Eca-109 cells. After 20 min of treatment, the cell survival rate was only about 20%, which was significantly better than treatment using PDT or PTT alone. The photosensitizer IR820 has good tissue penetration and excellent photothermal conversion efficiency, but it is easily metabolized in the body and cannot accumulate in large quantities in tumor sites. To overcome this problem, Liu et al. (2020) chose to deliver IR820 using hollow carbon nanocages (CNCs) and crosslink bovine serum albumin-manganese dioxide (BSA-MnO_2_) nanozyme on their surface to form the BMIOC nanosystem. In the BALB/c nude mouse esophageal cancer model, BMIOC not only rapidly produced O_2_ in the tumor tissue, enhancing the PDT efficacy, but also achieved high concentrations of IR820 at the tumor site; this system improved the PTT effect and realized real-time magnetic resonance (MR) imaging and NIR fluorescence imaging. Similarly, Chen et al. (2020b) used BSA-MnO_2_ as an oxygen generator and connected PDA encapsulated with porphyrin porous amine (PZM) to load the chemotherapeutic drug irinotecan to successfully prepare the FA-PDA@PZM/SN38@BSA-MnO_2_ (FA-PPSM) nanosystem for the treatment of esophageal cancer. The results showed significant tumor suppression effects of FA-PPSM under 580 nm and 808 nm laser irradiation. Compared to free irinotecan, the side effects, such as diarrhea and bone marrow suppression, were greatly reduced, achieving synergistic treatment of PTT, PDT, and chemotherapy.

## 8 Conclusion

NPs have potential value in improving the current diagnosis and treatment methods for esophageal cancer, showing definite effects in real-time imaging, drug delivery, radiosensitization, and photothermal and photodynamic therapy. Compared to traditional therapies, NPs have unique advantages in terms of targeted drug delivery and sustained release, improving drug pharmacokinetics, overcoming tumor cell resistance mechanisms, and reducing toxicity to normal tissues. However, there are still some key problems to be solved in order to realize clinical transformation. First, efficacy and safety are important evaluation indicators for the superiority of drugs. Currently, research on NPs is mainly focused on *in vitro* and animal experiments. However, existing animal models, including subcutaneous and orthotopic xenograft models based on tumor cell lines, patient-derived xenograft models (PDXs), and genetically engineered mouse models (GEMMs) (Gonzalez-Valdivieso et al., 2021), cannot fully simulate the physiological barriers between organisms and nanomedicines. The complexity of the human immune system makes the acute toxicity and potential long-term side effects of NPs difficult to predict. The Phase I clinical trial of the liposomal drug, MRX34, was terminated early due to severe immune-related adverse reactions occurring in 20% of patients (Hong et al., 2020). Secondly, the synthesis process of most NPs is complex, difficult, and relatively unstable for long-term preservation. It requires a significant investment of manpower, resources, and finances, which poses great challenges for pharmaceutical companies and quality control departments. How to optimize manufacturing processes and achieve large-scale and reproducible preparation while ensuring drug quality is a question that researchers must consider. In addition, the inherent pathways and mechanisms of NPs in tumor diagnosis and treatment have not been fully explored and elucidated. Only by further exploring key issues such as the interaction mechanism between NPs and the mononuclear macrophage system, the key conditions for NPs to be engulfed and taken up by tumor cells, and the environmental requirements for NPs to target tumor tissues, can we design nanosystems that are more suitable for clinical practical applications. Last but not least, the ethical issues brought about by new materials and new processes, including biological membranes, should not be ignored in the application of nanomedicine.

Therefore, in the process of translating nanomedicine from preclinical stage to clinical stage, multiple efforts are still needed. Currently, several anti-tumor nanomedicines have already been marketed or are in clinical research stages, mainly involving solid tumors, lung cancer, breast cancer, pancreatic cancer, etc. (Schmidt et al., 2020; Yoneshima et al., 2021; Kosaka et al., 2022; Rojas et al., 2023) However, there are still very few clinical trials specifically targeting esophageal cancer, and low effectiveness remains the main reason for trial failures. Thus, researchers need to further investigate from multiple disciplines such as materials science, physical chemistry, and pharmacology, taking into account key performance factors such as drug metabolism, biocompatibility, tissue distribution, and biological safety. They should design nanosystems that can overcome a series of biological barriers in the body, target tumor cells effectively and have good safety. More importantly, its effects were validated by developing novel animal models capable of simulating the heterogeneity and specific physiological environment of human tumors, accelerating clinical transformation. In addition, pharmaceutical companies need to establish quality standards and evaluation methods in advance during the mass production process, and strict implementation is ensured through collaboration between different operational units to guarantee product quality. At the same time, regulatory authorities should also develop relevant guidelines and identification technologies to promote the research and development process of nanomedicine. It is believed that in the near future, multifunctional NPs will provide a realistic basis for the integration of diagnosis and treatment, as well as individualized treatment of esophageal cancer, becoming another effective weapon for precise treatment of tumors.
